# Save your gut save your age: The role of the microbiome in stem cell ageing

**DOI:** 10.1111/jcmm.14373

**Published:** 2019-06-17

**Authors:** Yi Tan, Zongke Wei, Jiaoliu Chen, Junli An, Manling Li, Liuyun Zhou, Yanhua Men, Shan Zhao

**Affiliations:** ^1^ Obstetrics and gynecology department Dongguan nancheng hospital Dongguan China; ^2^ Shenzhen Rekindle Biotech Co., Ltd. Shenzhen China

**Keywords:** ageing, gut permeability, microbiome, stem cell

## Abstract

The tremendous importance of microbiota in microbial homoeostasis, alterations in metabolism and both innate and adaptive immune systems has been well established. A growing body of evidence support that dysbiosis or compositional changes in gut microbiota is linked to the ageing of stem cells in terms of dysregulations of metabolism, aberrant activation of the immune system as well as promoting epigenetic instability of stem cell. In this concise review, we elucidate recent emerging topics on microbiotic alterations and underlying mechanisms in stem cell ageing.

## 1 BACKGROUND

Ageing is an inevitable process in the majority of organisms. Ageing is associated with the occurrence of a spectrum of diseases including Alzheimer's disease, osteoporosis, type II diabetes and cancer. The problems of ageing and its related healthcare and economic issues result in a heavy burden all around the world.^1^, ^2^ Therefore, it is requisite to explore a novel strategy against ageing.

Stem cells are thought to demonstrate high potential in the field of anti‐ageing. During ageing, the decline in the self‐renewal and differentiation ability of stem cells and exhaustion of stem cells pool have been widely shown in various organs. Interference with ageing‐driven dysregulation on self‐renewal and differentiation ability of stem cells by remodulation of intrinsic and extrinsic factors should be a solution in the development of anti‐ageing drugs.

Factors contributing to stem cell ageing are classified as systemic and micro‐environment including hormones, systemic inflammation, host microbiome, local immune system and niche structure. Emerging studies suggest a close relationship between diversity, a constituent of host microbiota with the ageing process and ageing‐related diseases. For instance, skeletal mass, bone formation and bone growth are progressively declined with age, while modulation of gut microbiota by administration of antibiotics treatments in conventional mice or administration of specific‐gut microbiota in germ‐free mice results in a decrease and an increase bone mass and bone growth respectively. The molecular events underlying are commensal bacteria secreted short‐chain fatty acids to regulate the hormone for skeletal growth namely insulin‐like growth factor‐1.^3^ Another study also mentioned that disruption of microbiota interferes our normal circadian clock, consequently provoking metabolic disorders accompanied by exaggerating the ageing.^4^, ^5^ In mammals, the change in diversity of gut microbiota has been linked to the different age groups. In general, elderly peoples result less *Bacteroidetes* in terms of quantity and quality and more *Firmicutes* compared with healthy middle‐aged people In southwest China cohort.^6^ In experimental models, host microbiota has associated with the lifespan of Drosophila. Studies demonstrated that modulation of gut microbiota in drosophila by antibiotic treatment or stool transplantation from old Drosophila to young Drosophila increases or decreases the lifespan of *Dorosphila* respectively.^7^ However, how host microbiota affects the stem cell functions in term of ageing is still vague.

In this Review, we describe the contributions of host microbiota in stem cell ageing through modulation of metabolism, epigenetic changes as well as the inflammatory responses by the host immune system. We also introduce the possible microbiota‐mediated signalling pathways in stem cell ageing.

### 1.1 Host microbiome and metabolic changes in stem cell ageing

Ageing causes metabolic changes in stem cells. The metabolic changes in ageing stem cells contribute accumulation of mitochondrial damage accompanied with the imbalance between glycolysis and oxidative phosphorylation(OXPHOS) and accumulation of reactive oxygen species (ROS) resulting in depletion of stem cells pool.^8^

Metabolic changes in stem cell niches are attributed to the microbiota and its derived metabolites. A recent report has linked microbiota and haematopoietic stem cells(HSCs) differentiation via alteration of metabolic stress. The composition of gut microbiota is reconstituted by a high‐fat diet (HFD) in mice and alteration in gut microbiota leads to an increase the ratio of lymphoid cells to myeloid cells, indicating ageing haematopoiesis.^9^, ^10^ A similar phenomenon also exhibits in the intestinal stem cells. *Acetobacter pomorum,* a commensal bacterium residing in Drosophila, regulates host metabolic homoeostasis through insulin/insulin‐like growth factor signalling, resulting in enrichment of basal intestinal stem cells numbers.^11^ The possible mechanism for gut microbiota modulating host metabolism activity is gut microbial metabolites.

**Figure 1:**
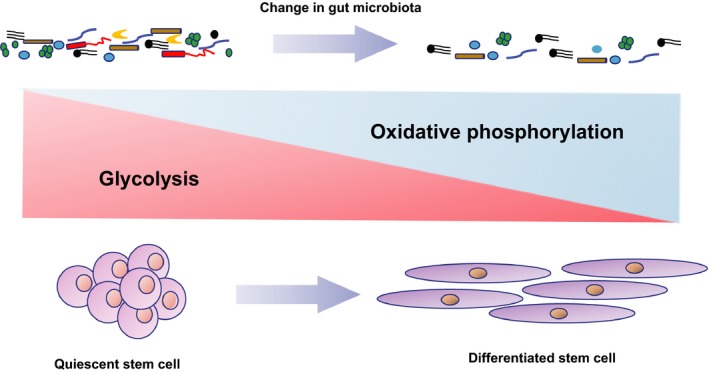
The metabolic programming of quiescent stem cells and differentiated stem cells in terms of the balance between glycolysis and oxidative phosphorylation. The common paradigm is that quiescent stem cells in the niche of normal commensal bacteria tend to prefer glycolysis accompanied with activation of anti‐oxidizing systems. On the contrary, differentiated stem cells under the niche of dysbiosis prefer oxidative phosphorylation rather than glycolysis to promote irreversible proliferation and differentiation of stem cells

One of the gut microbial metabolites is short‐chain fatty acid (SFCA) including acetate, butyrate and propionate.^12^ Under normal homoeostasis, a handful amount of SCFA improves the lifespan of the host. For example, Beta‐hydroxybutyrate (β‐HB) improves the lifespan of *C elegans* by suppressing histone deacetylase (HDAC) activity and activation of skinhead‐1(SKN‐1)/NF‐E2‐related factor (Nrf) pathway, subsequently facilitating the TCA cycle metabolism and ultimately increasing Forkhead box protein (FOXO) activity for stem cell proliferation.^13^ Nevertheless, under the conditions of “leaky” gut permeability caused by severe tissue damages and senescence, SFCA exerts their metabolic regulations on host stem cells through binding to G‐protein coupling receptors, subsequently suppressing insulin signalling and causing malfunctions of mitochondrial electron transport chain activity accompanied with the imbalance of NAD+/NADH ratio and dysregulation of NAD‐dependent deacetylase sirtuin‐1(SIRT1)/peroxisome proliferator activated receptor gamma coactivator 1 alpha(PGC1α) pathway.^14^, ^15^ As a result, more damaged mitochondria results along with an accumulation of ROS and imbalance between glycolysis and OXPHOS, eventually erroneous differentiation and proliferation of stem cells and in turn depletion of stem cell.^16^ Evidence in support of this notion comes from old HSCs expressed high OXPHOS levels as a result of dysfunctions in removing active mitochondria by impairing the autophagy process. This high levels of OXPHOS triggered the epigenetic modulations of old HSCs, subsequently promoted old HSCs undergoing myeloid differentiation and repressing the self‐renewal capacity (Figure 1)^17^. Moreover, aged Drosophila melanogaster exhibited stress caused‐ageing manifestations such as loss of tissues homoeostasis, hyperproliferation of intestinal stem cells as well as ageing‐associated intestinal dysplasia.^18^

Apart from SCFA‐triggered aberrant differentiation of stem cells and subsequent exhaustion of stem cells, SCFA also elicits their detrimental effects on the differentiation capacity of stem cells. For example, in intestinal epithelial stem cells, butyrate impedes colonic epithelial stem and progenitor proliferation through activating stress signalling pathway for FOXO3.^19^ In line with butyrate, another SFCA propionate also demonstrates the inhibitory effect on the differentiation capacity of human chorion‐derived mesenchymal stem cells (sMSCs)^20^. Reducing the differentiation capacity of stem cells is a requisite hallmark of ageing. In human mammary epithelial cells (HMEC), old progenitors demonstrated the reduced tendency of differentiation from HMEC to myoepithelial cells owing to impairment of Hippo pathway transducers Yes‐associated protein (YAP) and transcriptional co‐activator with a PDZ‐binding domain (TAZ).^21^ In agreement with HMEC, dysfunctions of autophagy in bone marrow‐derived mesenchymal stem cells result in accelerating ageing as exemplified by diminishing osteogenic differentiation and proliferation capacity, whereas autophagy activator rapamycin antagonizes the autophagy malfunction‐provoked senescence.^22^

Another type of microbial metabolites is endogenous ethanol. Bacterially derived ethanol is produced by Proteobacteria including *Escherichia coli* and other Enterobacteriaceae.^23^ The elevated endogenous ethanol causes the inhibitory effects on proliferating stem cells and depletion of stem and progenitor cells in the human hippocampus.^24^ Besides, more ethanol accumulates in the gut resulting in the increase of gut permeability by disruption of epithelial tight junctions especially zone occludins.^25^ This allows the translocation of pathogenic bacteria and its endotoxins and ethanol exert more directly destructive effects on tissues.^25^ As a consequence, depletion of stem cells pool occurs in order to compensate the damage tissues and accelerating ageing.

In *Drosophila*, the host microbiota also regulates the self‐renewal of stem cells in intestines for tissue regeneration. The possible mechanism for host microbiota‐mediated tissue regeneration is releasing of uracil, which further activates G‐protein‐coupled receptors of intestinal cells. Afterwards, ROS are released resulting in p38 and JNK signalling pathways activations and stimulate intestinal stem cells (ISC) proliferation.^26^ Interestingly, Janus‐faced of ROS has been reported in stem cell proliferation. Excessive activation of ROS stimulation can lead to activation of p38 and forkhead box protein signalling and eventually resulting aberrant stem cell differentiation.^27^ Taken together, an appropriate amount of ROS induces stem cell differentiation, but this process is under control by cellular homoeostasis to eliminate excessive ROS. Under ageing, the defects or delay of clearance of ROS, accumulation of ROS triggers the erroneous differentiation of stem cells and exhaustion of stem cells pool. In addition, quiescent haematopoietic stem cells with low levels of ROS accompanied with high levels of glycolysis and resemble young, had the capacity to eliminate excess ROS and ROS‐induced DNA damage, thus promoting the self‐renewal of stem cells and thwarting the aberrant proliferation and differentiation of stem cells, eventually maintaining the host in a younger condition.^28^, ^29^Reconstructing microbiome profiles restores the balance of ROS production in cellular level may pave the way for the development of anti‐ageing drugs. However, human gut contains more complex microbiome profiles; the role of human microbiota in ROS regulation still requires further investigations.

### 1.2 Host microbiome and epigenetic alterations in stem cell ageing

A growing body of evidence showed that modulation of global DNA methylation has been linked to ageing in various stem cells such as HSCs and other tissues.^30^, ^31^ Besides, muscle and haematopoietic stem cells exhibit the enhancement of histone repressive marks, such as histone H3 lysine 9 trimethylation (H3K9me3) and histone H3 lysine 27 trimethylation (H3K27me3) during the ageing process.^32^ Microbiota may exacerbate the ageing process by releasing more their metabolites butyrate in guts. Butyrate has known as an enhancer for H3K27me3 in vivo model.^33^ The possible underlying mechanism for enhanced H3K27me3‐modulated epigenetic modulation is increasing H3K27me3‐producing enzymes polycomb repressive complexes 2, subsequently facilitating the loss in epigenetic marking as well as drift of H3K27me3, diminishing the genes in glycolysis along with a decline of energy and NADH to NAD^+^ratio, thereby the stem cells become more susceptible to ROS and propel itself from quiescent state into an active state for differentiation and proliferation.^34^ Consistently, repressing the H327Kme3 in both *C elegans* and *D melanogaster* exhibited anti‐ageing effects.^35^, ^36^

Apart from histone modulation in gene expressions, microbiota also modulates host gene transcription by increasing specific transcription factors such as signal transducer and activator of transcription(STATs) and Interferon regulatory factors(IRFs) and facilitating their binding to target genes by opening nucleosome‐depleted *cis*‐regulatory regions in intestinal epithelial cells.^32^, ^37^ STATs is known as a regulator in myogenic differentiation for muscle tissues repair. Increased the expression of STATs transcriptional factors by microbiota resulting in reducing the reservoir of muscle stem cells and associated ageing diseases including Duchenne muscular dystrophy.^38^ On the contrary, pharmacological inhibition of STAT abrogated ageing triggered tissue loss by enhancing myogenic lineage progression and encouraging the muscle stem cell expansions.^39^Taken together, Ageing is linked to the regeneration capacity, function, self‐renewal of muscle stem cells. Overexpression and pharmacological inhibition of STAT‐signalling debilitate or boost the regenerative capacity of muscle stem cells respectively. Therefore, reconstruction of microbiota compositions in the gut may yield an anti‐senescent effect on the host by decreasing the intrinsic transduction pathway such as STATs as well as establishing favourable niches for muscle stem cells.

Moreover, another enhanced transcriptional factor IRFs has been reported in promoting senescence and impairing self‐renewal and differentiation of stem cells and consequently shorting lifespan in vivo.^40^ In an opposite way, depletion of microbiota reduces the relative methylation level of certain genes such as the toll‐like receptor 4 gene, which is a gene associated with chronic inflammation in ageing.^41^ Besides the responses of age‐associated genes STAT and IRFs are diminished in antibiotic‐treated mice.^42^ Collectively, microbiota involves in the regulation of age‐related gene expressions through facilitating transcription factors in the development of stem cells.

Moreover, DNA damage is an important hallmark for the ageing of stem cells. In the quiescent state, DNA damage is eliminated immediately by a self‐protective system such as autophagy. However, when senescence and influences of tremendous DNA stress exceed the removal capacity of stem cells, the DNA damage impedes the quiescence, self‐renewal, differentiation capacity of stem cells as well as exhaustion of stem cell pool through eliciting detrimental modulations on stem cell niches and circulatory environment (dysregulations of age‐associated signalling pathways as discussed below). Besides, such regulations provoke epigenetic alterations in DNA landscape of stem cells and further aggravate the senescence of stem cells by resulting in more DNA stress.^43^, ^44^ Emerging data underpin the involvement of microbiota in DNA‐mediated‐senescence modulation. A facultative anaerobe *E coli* produces genotoxin colibactin, which is a bacterial toxin leading to DNA damage, and subsequently enhancement of senescent‐associated microRNA (miR‐20a‐5p) suppresses p53 degradation and resulting in growth arrest of colonic epithelial cells.^45^ Metabolites of microbiota also participate in the DNA damage during ageing. Secondary bile acid deoxycholate is one of the most common metabolites produced by microbiota. Due to limitations of liver elimination, deoxycholate is accumulated in the liver and triggers DNA damage in gastric cardia stem cells and resulting in cellular senescence.^46^, ^47^ Not surprisingly, deoxycholate‐mediated senescent effects on HSCs are abrogated by administration of antibiotics mixture in obese mice, probably due to depletion of microbiota in guts.^48^

### 1.3 Host microbiome and immune system responses in stem cell ageing

In addition to modulating host metabolism and epigenetic alterations, host microbiome also affects local immune system during ageing.^49^ Loss of tissues homoeostasis as a result of reducing differentiation and proliferation capacity of stem cells is a manifestation of senescence. To date, a growing body of evidence has demonstrated immune cells, especially on regulatory T cells tremendously contribute to the tissues regeneration and stem cell niche. For instance, skin‐resident regulatory T cells recruit Notch ligand family member to elicit the differentiation and proliferation of hair follicle stem cells.^50^ Moreover, during the infection of Heligmosomoides polygrus, type 1 interferons (IFN)‐stimulating immune cells modulate the intestinal stem niche and reactivate the development of intestinal crypt for maintaining the tissues homoeostasis after injury.^51^

Emerging evidence supported that the alterations in the diversity of gut microbiota are bidirectionally regulated by immune cells. For example, the changes in compositions of gut microbiota such as increasing proteobacteria and decreasing in Faecalibacterium pranusnitizii, both of them are associated with ageing‐associated inflammatory diseases.^52^ Besides, natural killer T cells secrete IFN‐gamma dependent antimicrobial peptides to modulate the composition of gut microbiota by impeding the colonization of certain types of commensal bacteria.^53^ Intriguingly, 11 strains of commensal bacteria also regulate stimulations of IFN‐gamma dependent CD8 T cells yielding an anti‐cancer effect in the human intestine.^54^ Besides, microbiota metabolites such as SCFA (propionate, butyrate) also interfere with the balance between pro‐ and anti‐inflammatory mechanisms by modulations of regulatory T cells through forkhead box P3(FOXP3) signalling pathway.^55^

**Figure 2:**
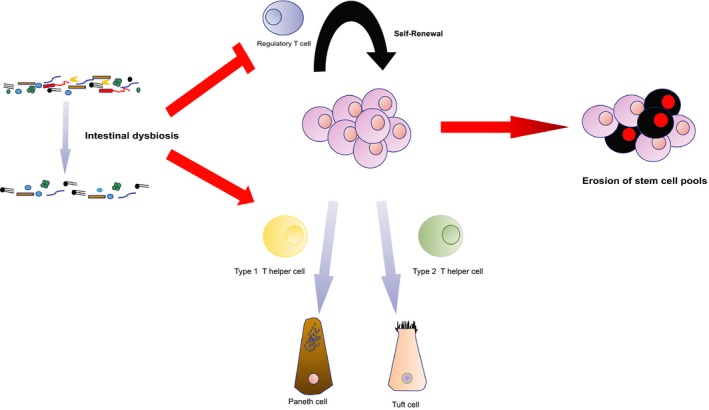
Control of intestinal stem cell homoeostasis through crosstalk between immune T cells and the intestinal commensal bacteria. In response to dysbiosis and increased gut permeability caused by ageing, T helper cells are activated to elicit the proliferation and differentiation of intestinal stem cells into Paneth cells and Tuft cells, accompanied with thwarting the functions of regulatory T cells which are responsible for promoting self‐renewal of intestinal stem cells. As a result, the intestinal stem cell pools are exhausted

Under the tissue homoeostasis, intestinal stem cells self‐renewal and differentiation are tightly regulated by regulatory T cells and T helper cells. Regulatory T cells stimulate Interleukin (IL), IL‐10 to promote the self‐renewal of stem cell. Upon the infection of Parasitic helminths and Salmonella enteric, T helper cells 1 (its cytokines IFN‐gamma) and T helper cells 2 (its cytokines IL‐13) promote the differentiation of intestinal stem cells to Paneth cell and Tuft cell respectively for combating the invasion of pathogens (Figure 2).^56^, ^57^ However, overreactive inflammatory responses provoke the over‐differentiation of stem intestinal stem cells to chemosensory cells, resulting in exhaustion of stem cell pool and accelerating senescence of the host. Consistently, an ageing‐associated decline of Bacteroides taxa triggers reductions of a low‐avidity mimotope to mimics islet‐specific glucose‐6‐phosphatase ‐catalytic‐subunit‐related protein (IGRP 206‐214). This mimotope is capable to recruit diabetogenic CD8+ T cell and subsequently inhibits overreactive inflammatory antigen‐presenting cells and further inflammatory responses.^58^ Apart from microbiota‐mediated dysfunctions of T cell homoeostasis in stem cells circuit, microbiota also influences T cell residence in secondary lymphoid organs and the proportion of residents T cells in secondary lymphoid organs increases with age.^59^ Recent studies have demonstrated that T cells participate in tissues regeneration in a mouse. It is possible that dysbiosis in the gut over a long period of time, it triggers the accumulation of T cells in “leaky” sites and stimulation a plenty amount of pro‐inflammatory cytokines and resulting in aberrant differentiation of stem cells and depletion of the stem cells’ reservoir, eventually the exhaustion of stem cell pool causes accelerating senescence.

Microbiota not only regulates the immune cell homoeostasis but also beget the increasing the gut permeability during ageing. Concerning gut permeability, tight junction proteins such as occludin and claudin are vital markers in barriers functions in intestinal tissues.^60^ Emerging evidence demonstrated that increasing gut permeability as expressed by diminishing the expressions of tight junction proteins, leading to more microbiota and their metabolites pass through the gut lumen, subsequently activation of immune responses such as macrophages, dendritic cells and T cells. Activated immune cells stimulate several inflammatory cytokines such as tumour necrosis factor (TNF), IL‐10 and IL‐17 against ‘leaky’ gut microbiota.^61^ Afterwards, the pro‐inflammatory responses affect stem cell niches, self‐renewal, differentiation capacity of stem cells.^62^, ^63^ For instance, Both TNF and bacterial metabolites lipopolysaccharide(LPS) have been reported in self‐renewal of hepatic stem cells for liver regeneration through activation of Toll‐like receptor 4 and IL‐6 and TNF‐α.^64^ In the bone, both IL‐6, IL‐10 and TNF‐ α have shown a positively associated with the differentiation of bone marrow stem cells.^65^ In the skin grafts, IL‐17 promotes the self‐renewal and differentiation of mesenchymal stem cells.^66^ In line with this hypothesis, alterations in gut microbiota attenuate the systemic and hepatic inflammation in obese and diabetic mice by restoring endogenous intestinotrophic proglucagon‐derived peptide‐dependent modulations of the gut permeability.^67^

Collectively, reconstruction of gut microbiota through the administration of probiotics restores the balance of microbiome in guts and ameliorates the gut permeability and preventing pathological activation of immune responses and its driven abnormal differentiation of stem cells in the recovery of hepatic functions.^68^ However, most of the publications for immune system‐mediated stem cell proliferation are under the condition from injury or diseases caused cell regeneration by stem cell. The effects of microbiota in normal conditions are poorly understood and require further investigations.

### 1.4 Host microbiome and ageing‐associated developmental pathway

Host microbiome leads ageing‐associated development on stem cells through several pathways including Wnt, Notch, transforming growth factor beta(TGF‐ β), mitogen‐activated protein kinases (p38‐MAPK) and JUN N‐terminal kinase(JNK) signalling pathways.

### 1.5 Wnt signalling pathway

Activation of Wnt signalling pathway has been linked to the stem cell ageing in muscle stem cells (MuSCs) and HSC by promoting aberrant differentiation and impairing the interaction of stem cells with the microenvironment and eventually loss of self‐renewal activity and differential capacity of stem cells.^69^, ^70^ Microbiota may exert the pro‐ageing effect on stem cells through their metabolites and activation of Wnt signalling. Butyrate is one of most common metabolites from commensal bacteria in the gut. Butyrate promotes the expansion of cancer stem cells (CSCs) through accumulating Wnt‐signalling substrate β‐catenin and subsequently increasing expression of WNT reporter activity by its HDAC inhibitor characteristic.^71^ As shown in Table 1, the functions of SCFA in modulating various stem cells and the underlying mechanisms are summarized. The Besides, another bacterial metabolite, lactic acid, produced by Bifidobacterium and Lactobacillus spp Lactic acid targets G‐protein‐coupled receptor 81 on intestinal stem cells and promotes stem cells proliferation through Wnt‐3/ beta‐catenin signalling pathways. Consistently, administration of lactate ameliorated radiation‐ and chemotherapy‐ triggered intestinal damage which can be repaired by ISCs.^72^

**Table 1:** The functions of short chain fatty acid in modulating various stem cells and the underlying mechanisms

Type of short chain fatty acid	Host	Type of stem cells	Effects on stem cells	Underlying mechanisms	Reference
Beta‐ hyroxybutyrate	Caenorhabditis elegans	Not mentioned	Profileration	Activation of SKN‐1‐ Nrf‐FOXO	13
Propionate, butyrate	Mice	Intestinal stem cells	Profileration	Acitvation of Foxp3	55
Butyrate	Mice	Not mentioned	Profileration	Activation of IGF‐1	3
Butyrate	Mice	Intestinal stem cells	Self‐renewal	Acitvation of FOXO3	19
Propionate	Human	Mescenchymal stem cells	Self‐renewal	Acitvation of FFAR2‐PPARγ	20
Butyrate	Human	Cancer stem cells	Self‐renewal	Activation of Wnt‐signalling	71
Butyric acid	Porcine	Mesenchymal stem cells	Profileration	Acitvation of PPARγ‐CCAAT signalling	73
Indole‐3‐acetic acid	Human	Dental pulp stem cells	Self‐renewal	Activation of Akt‐Nrf‐HO‐1 pathway	74
Gelatin‐hydroxyphenyl propionic acid	Human	Mesenchymal stem cells	Profileration	N/A	75
Butyrate	Mice	Colonic epithelial stem cells	Self‐renewal	Inhibition of the activity of histone deacetylases	76
Poly‐3‐hydroxybutyrate	Rat	Mescenchymal stem cells	Profileration	N/A	77
Acetate	Human	Multipotent adipose tissue‐derived stem	Profileration	Inhibition of the activity of hormone‐sensitive lipase	15
Sodium butyrate	Human	Adipose tissue‐derived mesenchymal stem cells	Self‐renewal	Inhibition of the activity of histone deacetylases	78
Sodium butyrate	Human	Amniotic membrane‐derived mesenchymal stem cells	Self‐renewal	Activation of ERK‐1	79
Butyrate	Human	Colonic epithelial stem cells	Self‐renewal	suppression of Kruppel‐type zinc‐finger family transcription	80
Metformin‐butyrate	Human	Breast cancer stem cell	Self‐renewal	activation of AMPK	81
Butyric acid	Human	Adipose‐derived stem cells	Profileration	acitvation of PPARγ signalling	82

Besides the impacts of microbial metabolite on the gut, another IFN, which is a product in the gut with the assistance of microbiota, activates β‐catenin activity and causes abnormal proliferation of intestinal epithelial cells as well as an increase in the expression of cellular senescence marker p21, and leading ageing‐associated diseases.^83^

### 1.6 Notch and TGF‐beta signalling pathways

The Notch signalling pathway is a key regulator to control intestinal epithelium homoeostasis by modulation of self‐renewal rate of stem cell and differentiation rate of progenitor cells into various intestinal cell types such as paneth cells, goblet cells.^84^ Ageing has been recognized as a suppressor in Notch signalling pathway by diminishing the regenerative capacity of stem cells and subsequent recruitment of cyclin‐dependent kinase inhibitors such as p16, which is an activator in cellular senescence.^85^, ^86^, ^87^ Microbiota may suppress Notch signalling pathway. In bone marrow mesenchymal stem cells (BMSCs), gut dysbiosis resulting from hypoxia causes induction of D‐galactose accumulation and increasing the expressions of the p16 cellular level and leading senescence of BMSCs through Notch pathway.^88^

Apart from suppressing Notch signalling pathway, microbiota also influences the TGF‐β. TGF‐ β is an inducer of quiescence to maintain HSC pool and prevent exhaustion of HSC pool and maintain the niche of HSC.^89^ During ageing, the abundance of gut microbiome phylum *Bacteroides* decreases and diminishes the *Bacteroides*‐driven induction of TGF‐β through hindering the regulatory T cells differentiation.^90^ It is possible that a decline in TGF‐β expression contributes to the disruption of quiescence state of HSC and in turn aberrant differentiation of stem cells and ultimately resulting in erosions of HSC functions.

### 1.7 p38‐MAPK signalling pathway

Ageing associated activation of p38‐MAPK and suppression of Fibroblast growth factor 1(FGF1) signalling pathways impair the self‐renewal and regenerative capacity of stem cells such as MuSC.^91^ Microbiota contributes to the proper control of stem cell activity through p38‐ MAPK pathways. In neural progenitor or radial glial cell, gut microbiota stimulates small molecules and they translocate to the brain and influencing the stage and region‐specific of the striatum, subsequently regulating TGF‐β and p38 pathway through sphingosine‐1‐phosphate to maintain brain stem cell development and functions.^92^, ^93^

Another ageing‐associated phenotype, tissues degeneration has been shown regarding enhancement of *proteobacteria*. From the Italian and Chinese cohort, gut microbiome *proteobacteria* have been enriched in short‐living participants.^94^ The potent mechanism for *proteobacteria* hindering tissue regeneration is activation of transforming growth factor beta‐activated kinase 1 (TAK1)‐mitogen activated protein kinase kinase (MKK)‐p38‐MAPK signalling pathway.^95^

### 1.8 JUN N‐terminal kinase stress‐related signalling pathway

Ageing stem cells have been shown in an increase of JNK signalling, which promotes the aberrant proliferation and differentiation of intestinal stem cells, and in turn hyperplasia of epithelial cell areas and increasing in gut permeability.^96^ In Drosophila, commensal bacteria inside the host control over renewal capacity of stem cell activity through activation of both JAK‐STAT (Janus kinase‐signal transducers and activators of transcription) and JNK pathway in the context of with or without invasive bacterial infection.^97^ Consistently, depletion of commensal bacteria such as dysbiosis in *D melanogaster* also results in JNK signalling activation and further leading to erroneous differentiation of ISCs during ageing.^18^

Collectively, microbiota regulates JNK pathway not only for self‐renewal of epithelium cells, albeit very minor compared with under condition of invasive infections from other bacteria, but also rising the defensive responses against bacterial invasion. Furthermore, JNK pathway is indispensable for ISC regeneration. Commensal bacteria such as dysbiosis in *D melanogaster* also results in JNK signalling activation and further leading to erroneous differentiation of ISCs during ageing.^18^ In Drosophila, dysregulation of JNK contributes to the accumulation of mis‐differentiated ISC daughter cells and in turns resulting in loss of tissue homoeostasis and ageing.^98^

## 2 CONCLUSIONS AND FUTURE PERSPECTIVES

The role of stem cells in ageing is repairing damaged tissues and regenerate specific tissues in order to maintain tissues homoeostasis. During ageing, dysregulation of metabolism, alterations in immune system, epigenetic modulations, erroneous regulation of extrinsic signalling pathways lead to the accumulation of stress responses and impairing the functions of stem cells.

Increasing evidence suggests that microbiota plays an important role in stem cell ageing. In line with this hypothesis, dysbiosis in the gut triggers serious responses. Dysbiosis causes an increase in gut permeability and facilities the commensal metabolites, derived small molecules and stress responses entering the blood circulation system and delivering ‘leaky’ materials to the multiple tissues. Afterwards, metabolites influence on stem cells metabolism, activation of pro‐inflammatory cytokines in the immune system as well as induction of DNA damage and aberrant methylation of histone mark in stem cell epigenetics.

During this process, the ‘leaky’ microbiota and their metabolites activate Notch, Wnt, TGF‐ β along with JNK pathways causing the erroneous differentiation of stem cells and overuse of stem cells thus resulting loss of tissue homoeostasis and ageing in the long‐term. At the same time, the host becomes susceptible to bacteria or virus‐mediated tissues damages due to the increase in the gut permeability and subsequently more stem cells are required for tissues repairing and thus resulting in the positive feedback loop on exhaustion of stem cell pool and accelerating the ageing process.

Reconstruction of gut microbiota and in turn restoring gut permeability is a crucial strategy in dealing with the stem cell‐associated ageing process. To date, faecal microbiota transplantation brings our attention to the reconstruction of gut microbiota. There is evidence that faecal microbiota transplantation improves the growth performance by reducing intestinal permeability in piglets and maintaining intestinal morphology.^99^ Since increasing intestinal permeability has been known in ageing‐associated diseases, restoring intestinal permeability by faecal microbiota transplantation may be an effective and regenerative medicine strategy in stem cell development for aged. However, it remains to be investigated whether fecal microbiota transplantation from young donors to old receivers restores the self‐renewal, differentiation and regenerative capacity of stem cells in order to ameliorate the health span. In conclusion, more investigations in the cross‐talk between microbiota and intestinal stem cells are required to pave the way for the development of therapeutic drugs in prolonging lifespan and tackling ageing‐associated diseases.

## CONFLICT OF INTERESTS

The authors declare that they have no competing interests.

## AUTHORS' CONTRIBUTIONS

YT and SZ performed the literature research, drafted the manuscript andmade the figure; JA, ZK and LZ revised the manuscript and directedthe review to be more focused, (JC contributed equally tothis work); HM made the table; ML, YT and SZ gave the final approval for the article to be published.
